# Isolated medial antebrachial cutaneous nerve injury after blunt trauma: a case report

**DOI:** 10.1186/s13256-023-03797-1

**Published:** 2023-03-13

**Authors:** Zahra Babaeian, Alireza Ashraf, Fariba Erfani

**Affiliations:** 1 Student Research Committee, Department of Physical Medicine and Rehabilitation, Shiraz University of Medical Sciences, Shiraz, Iran; 2 Department of Physical Medicine and Rehabilitation, Shiraz Geriatric Research Center, Shahid Faghihi Hospital, Shiraz University of Medical Sciences, Karimkhan Zand Street, Shiraz, 71348-44119 Iran

**Keywords:** Blunt trauma, Electrodiagnosis, Medial antebrachial cutaneous nerve injury, MAC nerve injury

## Abstract

**Background:**

The medial antebrachial cutaneous nerve is a branch of the brachial plexus that contains C8–T1 segments. Injury of this nerve by various mechanisms has been reported in the literature; however, currently, there is no reported case of medial antebrachial cutaneous nerve injury in the setting of acute blunt trauma.

**Case presentation:**

This case report presents the case of a 34-year-old Persian female with dysesthesia and pain in the medial side of the forearm immediately following a blunt trauma by mechanism of elbow external rotation. On electrodiagnostic evaluation, the medial antebrachial cutaneous nerve sensory nerve action potential of the symptomatic side had a significant amplitude drop (more than 50%), compared with the other side. On follow-up electrodiagnosis, after several sessions of physical therapy, the medial antebrachial cutaneous nerve sensory nerve action potential still had a significant amplitude difference.

**Conclusion:**

Blunt trauma can be one of the causes of medial antebrachial cutaneous nerve involvement. An electrodiagnostic study can be helpful in the diagnosis of this nerve injury after blunt trauma.

## Background

The medial antebrachial cutaneous (MAC) nerve is a branch of the brachial plexus that carries fibers of C8–T1 segments [1, 2]. It is responsible for the medial side of the forearm and olecranon skin sensation [3, 4]. In the literature, MAC nerve involvement has been reported to have various mechanisms. We present a case of acute blunt trauma-induced injury to the MAC nerve that was diagnosed by a nerve conduction study. To the best of our knowledge, this is the first time that this nerve injury has been reported after acute blunt trauma.

## Case presentation

A 34-year-old right-handed Persian female engineer was referred to the electrodiagnostic clinic due to dysesthesia and pain in the medial side of her right hand and forearm. She had a history of blunt trauma to her right forearm about 40 days before the first evaluation. The mechanism of trauma was an external rotation of the elbow. Abruptly, she developed lancinating pain and dysesthesia in the medial side of the forearm. After 2 days, the nature of the pain became dull. Also, paresthesia, tingling, and numbness started from the medial side of the elbow, to the hand and the fifth finger. She complained of difficulty in writing due to this annoying dysesthesia. There was no complaint of weakness in the affected limb. She was nulliparous. In her past medical history, she did not have any significant social, environmental, or drug history prior to diagnosis. She denied alcohol consumption or smoking. She did not have polyneuropathy, chronic systemic disease, phlebotomy, injection, or surgical intervention at the elbow. Also, there was no significant psychological disorder or related family history.

On physical examination, she seemed well nourished with a blood pressure of 115/80 mmHg, pulse rate of 75 beats per minute, and axillary temperature of 36.2 °C at the first outpatient visit. The light touch and pinprick sensation were impaired on the medial side of the right forearm. Range of motion, manual muscle testing, and deep tendon reflexes were normal. Hoffmann’s and Babinski signs were negative. Mild tenderness in the anteromedial part of the elbow was detected. There was no Tinel’s sign around the elbow region.

**Fig. 1:**
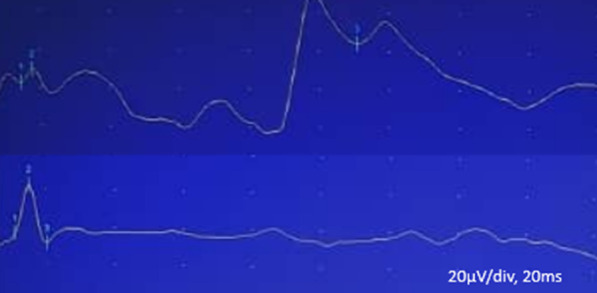
Nerve conduction responses of both sides of the medial antebrachial cutaneous nerve. Lower trace: normal response obtained from the left side medial antebrachial cutaneous, peak latency 1.55 milliseconds, amplitude 26.9 µV. Upper trace: abnormal response obtained from the right side medial antebrachial cutaneous, peak latency 1.77 milliseconds, amplitude 7.6 µV (more than 50% amplitude drop compared with the other side)

On nerve conduction study (NCS), sensory nerve action potential (SNAP) of the median (third finger), ulnar (fifth finger), radial (snuff box), and dorsal ulnar cutaneous nerves had normal peak latency and amplitude, without a significant difference to the asymptomatic side. On further evaluation, the medial antebrachial cutaneous nerve SNAP of the symptomatic side had a considerable amplitude drop (more than 50%) compared with the other side (as shown in Fig. 1 and Table 1). Also, compound nerve action potential (CNAP) of the ulnar nerve across the elbow by stimulating the wrist and recording above the elbow showed mild conduction block on the right side compared with the left.

**Table 1:** The data of the first nerve conduction study of the case

Nerve/site	Onset latency (milliseconds)	Peak latency (milliseconds)	Amplitude (µV)
Right ulnar CNAP	3.02	3.85	40.9
Left ulnar CNAP	3.02	3.65	70.5
Right MAC	1.46	1.77	**7.6**
Left MAC	1.09	1.51	26.9

*µV* microvolt, *CNAP* compound nerve action potential, *MAC* medial antebrachial cutaneous

Motor NCS of the median and ulnar nerves was normal. F-wave of the abductor digiti minimi was normal. On needle electromyography of the right flexor carpi ulnaris and first dorsal interosseous, there was normal motor unit action potential (MUAP) and recruitment without spontaneous activity. In summary, this study showed isolated mild right medial antebrachial cutaneous nerve injury.

The right elbow X-ray was normal. Magnetic resonance imaging of the right elbow revealed faintly visualized signal changes in the proximal and posterior aspect of the medial collateral ligament (MCL) with no definite evidence of defect or tear. A thin wall cyst with the same signal to synovial fluid just lateral to the olecranon was seen connecting to the joint space measuring 9 × 5 × 2 mm incidentally.

On follow-up electrodiagnosis after 1 month, the conduction block in the right ulnar CNAP resolved, but the MAC nerve SNAP still had a significant amplitude difference (Table 2). She did not have any hospital admission or drug prescription. However, she had undergone several sessions of physical therapy during this period. This course of physical therapy included mobility of the elbow and wrist, stretching of forearm muscles, nerve gliding exercises for the ulnar nerve, and transcutaneous electrical nerve stimulation. Numbness and sensory complaints of the medial side of the right hand and fifth finger showed improvement, but the sensory disturbance remained at the medial side of the forearm until 6 months follow-up.

**Table 2:** The data of the second nerve conduction study of the case (1 month later)

Nerve/site	Onset latency (milliseconds)	Peak latency (milliseconds)	Amplitude (µV)
Right ulnar CNAP	3.33	4.06	58.6
Left ulnar CNAP	3.18	3.85	61.4
Right MAC	1.72	2.08	**11.9**
Left MAC	1.51	1.93	18.9

*µV* microvolt, *CNAP* compound nerve action potential, *MAC* medial antebrachial cutaneous

## Discussion

In this case report study, we presented the case of a 34-year-old female with isolated MAC nerve injury after blunt trauma.

The medial antebrachial cutaneous nerve originates from the medial cord of the brachial plexus in continuation of the lower trunk. The MAC nerve contains the fibers of C8 and T1 nerve roots [1, 2]. It descends through the brachial fascia along with the basilic vein, brachial artery, and median and ulnar nerves [5]. At about 10 cm proximal to the medial epicondyle, it is divided into two branches (anterior and posterior) and continues to the wrist. It is a pure sensory nerve that innervates the anteromedial part of the distal arm, antecubital fossa, posterior olecranon region, and medial volar aspect of the forearm. Different variations were reported in the anatomical course of this nerve [2, 6, 7]. In the literature, some reported causes of MAC nerve involvement include brachial plexopathy [8] and thoracic outlet syndrome [9]. This nerve involvement was also reported with tuberculoid leprosy neuritis [10] and subcutaneous lipoma [11]. There are some iatrogenic causes, including steroid injection due to medial epicondylitis, routine venipuncture, cubital tunnel surgery, loose body removal, elbow arthroscopy, open fractures fixation, tumor excision, panniculitis excision, brachial plexus block, and arthrolysis [12–20]. In one case report, it occurred after repetitive minor trauma [5]. Injury of the MAC nerve occasionally occurred due to iatrogenic reasons during the interventions. To the best of our knowledge, this is the first injury of MAC nerve with blunt trauma with elbow external rotational mechanism. Because damage to this nerve rarely occurs, its evaluation may be missed in routine electrodiagnostic studies. Although spontaneous recovery of this nerve may be possible, the delay in timely diagnosis can cause imposing unnecessary diagnostic work-ups to evaluate other differential diagnoses of forearm dysesthesia. It may seem that MAC nerve injury has no important role in daily activity, but in this case, it interfered with her work-related activities, such as writing for extended periods. It affected her quality of life. Although spontaneous recovery of this nerve is possible, appropriate treatment could be administered promptly to assist the patient in early recovery. As a result, the patient would have the opportunity to conveniently return to work and routine daily life. Aiming to control the symptoms, we started conservative management for her. Physical therapy, including nerve gliding exercises, was done that was relatively effective, especially on the ulnar nerve block at the elbow.

## Conclusion

Blunt trauma can be one of the causes of MAC nerve involvement. Because this nerve is not evaluated in routine electrodiagnostic study, damage to this nerve may be missed. It is recommended that this nerve be evaluated in any patient who presents with any sensory complaint in the medial side of the forearm and wrist.

## Data Availability

Not applicable.

## Abbreviations

MAC: Medial antebrachial cutaneous
NCS: Nerve conduction study
SNAP: Sensory nerve action potential
CNAP: Compound nerve action potential
MUAP: Motor unit action potential
MCL: Medial collateral ligament
µV: Microvolt

## References

[1] Stylianos K, Konstantinos G, Pavlos P, Aliki F (2016). Brachial branches of the medial antebrachial cutaneous nerve: a case report with its clinical significance and a short review of the literature. J Neurosci Rural Pract.

[2] Benedikt S, Parvizi D, Feigl G, Koch H (2017). Anatomy of the medial antebrachial cutaneous nerve and its significance in ulnar nerve surgery: an anatomical study. J Plast Reconstr Aesthet Surg.

[3] Polcaro L, Charlick M, Daly DT. Anatomy, head and neck, brachial plexus. StatPearls. 2021. 30285368

[4] Thomas K, Sajjad H, Bordoni B. Anatomy, shoulder and upper limb, medial brachial cutaneous nerve. StatPearls. 2021. 30969651

[5] Yildiz N, Ardic F (2008). A rare cause of forearm pain: anterior branch of the medial antebrachial cutaneous nerve injury: a case report. J Brachial Plex Peripher Nerve Inj.

[6] Race CM, Saldana MJ (1991). Anatomic course of the medial cutaneous nerves of the arm. J Hand Surg.

[7] Ballard T, Smith T. Anatomy, medial antebrachial cutaneous nerve. StatPearls. 2020. 31869102

[8] Seror P (2004). Medial antebrachial cutaneous nerve conduction study, a new tool to demonstrate mild lower brachial plexus lesions. A report of 16 cases. Clin Neurophysiol.

[9] Machanic BI, Sanders RJ (2008). Medial antebrachial cutaneous nerve measurements to diagnose neurogenic thoracic outlet syndrome. Ann Vasc Surg.

[10] Martins R, Siqueira M, Carvalho A (2004). A case of isolated tuberculoid leprosy of antebrachial medial cutaneous nerve. Neurol Sci.

[11] Seror P (1993). Forearm pain secondary to compression of the medial antebrachial cutaneous nerve at the elbow. Arch Phys Med Rehabil.

[12] Yildiz N (2014). Medial antebrachial cutaneous neuropathy in a teacher: a case report. Research.

[13] Richards R, Regan W (1989). Medial epicondylitis caused by injury to the medial antebrachial cutaneous nerve: a case report. Can J Surg.

[14] Horowitz SH (1994). Peripheral nerve injury and causalgia secondary to routine venipuncture. Neurology.

[15] Asheghan M, Khatibi A, Holisaz MT (2011). Paresthesia and forearm pain after phlebotomy due to medial antebrachial cutaneous nerve injury. J Brachial Plex Peripher Nerve Inj.

[16] Lowe JB, Maggi SP, Mackinnon SE (2004). The position of crossing branches of the medial antebrachial cutaneous nerve during cubital tunnel surgery in humans. Plast Reconstr Surg.

[17] Sarris I, Göbel F, Gainer M, Vardakas DG, Vogt MT, Sotereanos DG (2002). Medial brachial and antebrachial cutaneous nerve injuries: effect on outcome in revision cubital tunnel surgery. J Reconstr Microsurg.

[18] Kelly EW, Morrey BF, O'Driscoll SW (2001). Complications of elbow arthroscopy. JBJS.

[19] Chiu Y, Huang Y, Chang C (2008). Medial antebrachial cutaneous neuropathy: a case report. Electromyogr Clin Neurophysiol.

[20] Jung MJ, Byun HY, Lee CH, Moon SW, Oh M-K, Shin H (2013). Medial antebrachial cutaneous nerve injury after brachial plexus block: two case reports. Ann Rehabil Med.

